# Enhancing University Students’ Motivation in Basketball Courses through Tactical Games Model

**DOI:** 10.3390/bs14070515

**Published:** 2024-06-21

**Authors:** Jiaxu Wang, Chen Soon Chee, Shamsulariffin Samsudin

**Affiliations:** Department of Sports Studies, Faculty of Educational Studies, Universiti Putra Malaysia, Serdang 43400, Malaysia; gs62326@student.upm.edu.my (J.W.); shamariffin@upm.edu.my (S.S.)

**Keywords:** tactical games model, motivation, university students, basketball course

## Abstract

As the COVID-19 pandemic subsides, universities face challenges such as diminished student physical fitness and a decreased interest in physical education courses. The purpose of this study was to evaluate the effectiveness of the tactical games model (TGM) in enhancing university students’ motivation in basketball courses, using a comparison control group taught using the direct instruction model (DIM). Additionally, this research delves into the motivational dynamics explained by self-determination theory, aiming to identify key factors influencing student engagement and participation. A total of 141 sophomore university students were analyzed and divided into an experimental group (68 students) and a control group (73 students). The participants engaged in an 8-week teaching intervention program. To assess motivation, the Sport Motivation Scale-II (SMS-II) was administered both before the start and one week after the conclusion of the intervention. Differences in motivation and subscale scores between the TGM and DIM groups were evaluated using analysis of variance (ANOVA) and analysis of covariance (ANCOVA). The results of the study demonstrated that the TGM significantly enhanced university students’ motivation (SDI: F = 6.949; *p* = 0.009; η² = 0.049). Furthermore, TGM enhanced scores on intrinsic and extrinsic motivation sub-scales more effectively than the DIM. These findings advocate for the adoption of TGM by university instructors as a potent tool to elevate student motivation, emphasizing the importance of focusing on both intrinsic and extrinsic motivational elements within physical education programs.

## 1. Introduction

Physical activity is crucial in health and wellness, offering extensive physiological and psychological benefits [1]. Physiologically, it aids in weight management and promotes overall well-being [2,3,4]. Psychologically, it facilitates stress reduction, stabilizes emotions, ameliorates unfavorable psychological conditions, and decreases the prevalence of mental disorders [5,6,7]. Currently, students’ psychological well-being globally is increasingly compromised by the mounting pressures of academic work. The onset of the COVID-19 pandemic in late 2019 severely disrupted daily routines and has led to significant life disruptions. This prevented students from attending school and participating in outdoor physical activities due to widespread school closures and government-imposed restrictions. Consequently, these restrictions have markedly reduced students’ physical well-being [8,9,10,11]. Though the COVID-19 pandemic is primarily considered to have ended, its effects on students persist. According to the Esports [12] report, the surge in the video game industry has prompted a shift in students’ leisure preferences, with many opting for gaming as their primary recreational activity. This preference for gaming suggests a tendency to remain indoors rather than engage in physical activities. This shift has resulted in decreased interest and participation in sports activities. Consequently, there is an urgent need to implement strategies that increase students’ engagement with physical activities, which are crucial for their mental and physical health improvement.

Since the 20th century, numerous researchers have identified “motivation” as a pivotal factor influencing individual decisions to engage in various activities [13,14,15]. Motivation can be conceptualized as an internal force, a psychological drive, or a manifestation of personal needs. Importantly, positive motivation is vital in encouraging individuals to pursue and realize their aspirations and goals [16,17,18,19,20]. In the realm of education, motivation is regarded as a crucial factor that shapes students’ learning outcomes [21,22]. Similarly, in physical education, motivation is integral in determining students’ participation levels in the activities offered, reflecting its broad impact on educational engagement [23,24,25]. Positive motivation is a key driver of student participation in physical education and sports activities, likely encouraging the development of regular physical activity habits [26,27,28,29].

Psychologists Edward Deci and Richard Ryan introduced the concept of basic psychological needs, central to the theory of autonomy, competence, and relatedness (TAD), commonly known as self-determination theory (SDT). This theory suggests that human motivation is fueled by fulfilling three intrinsic psychological needs: autonomy, competence, and relatedness. These needs are universal, transcending various cultures and age groups. Meanwhile, self-determination theory (SDT) provides a robust, scientifically validated theory that explains students’ motivations for engaging in physical education programs. This theory has become the most extensively adopted in physical education research [30,31,32,33,34,35]. Under SDT, enhanced motivation is believed to deepen students’ understanding and increase their participation in educational activities [36,37]. Furthermore, suppose students experience strong engagement and achievement during learning activities (such as winning a game by scoring points or independently resolving a challenge during play). In that case, their motivation will likely be further invigorated [38]. Moreover, some scholars believe intrinsic motivation directly influences student participation in academic courses, as it is a crucial determinant of their psychological and educational engagement [39,40]. Therefore, a greater emphasis on enhancing students’ intrinsic motivation is essential to boost interest and participation in basketball courses.

Physical education courses in schools play a crucial role in encouraging students to engage in physical activities. Schools offer students professional venues for various sports along with a diverse array of physical education programs [41,42,43]. High-quality instruction in these programs not only aids in the acquisition of knowledge and skills but also enhances students’ psychological and emotional well-being, fostering a lifelong commitment to physical activity [44,45]. However, numerous researchers have identified teaching methods as primary determinants of the quality of students’ learning experiences in physical education courses [46,47,48]. Despite the variety of approaches, most educators continue to employ traditional approaches. Such conventional techniques often fail to provide students with satisfactory learning experiences or achieve the intended educational outcomes [49,50,51,52]. Specifically, basketball teachers at Chinese universities predominantly employ the traditional direct instruction model (DIM) to enhance students’ skills and tactical understanding. Typically, a course begins with a review session where students recapitulate the previous lesson’s content. This is followed by introducing and explaining new skills or tactics, and students then dedicate significant time to practice these skills or tactics extensively. The session typically concludes with a period of free activities or games [53,54,55]. While the direct instruction model (DIM) can effectively develop students’ basketball skills, it isolates skill learning from real-game contexts, which are inherently competitive [56]. Learning skills in such a non-competitive environment may lead students to view basketball as a complex and challenging sport. The isolated instruction of skills, tactics, and rules might overwhelm students, making the sport seem less accessible and more tedious. Such a situation can complicate the learning process, potentially leading to disinterest and boredom in basketball courses [52,53,57,58,59,60].

Among the many pedagogical approaches in physical education, the game-centered approach (GCA), introduced by Bunker and Thorpe in 1982 [61], is a recognized pedagogical strategy in physical education, embraced by both teachers and researchers. This approach utilizes the intrinsic allure of games to boost student engagement and motivation [62,63,64], addressing common educational challenges such as student interest and learning effectiveness [65,66,67]. The inclusion of games in the curriculum mirrors the growing trend of GCA, a strategy that has become widespread in contemporary teaching practices [68,69,70,71]. Incorporating GCA into the physical education curriculum effectively includes participants of all ages and abilities while increasing their enjoyment and interest in physical activities [72,73,74].

The tactical games model (TGM) is one of the dominant pedagogical models in many GCAs. It was introduced into the U.S. physical education curriculum by Griffin, Linda L. and Mitchell in 1997 with their adaptation of the teaching game for understanding (TGFU) [73,74]. Unlike the teaching game for understanding (TGFU) [75], where the entire game is typically introduced at the end of the course, the tactical games model (TGM) centers the course around the game itself, focusing on actively engaging the students in the game as the center of the course. This model simplifies the original six-stage process of TGFU into three streamlined stages: (1) starting with an exaggeratedly modified game that aligns with specific instructional objectives; (2) developing tactical awareness through tactical problems that prompt students to understand necessary technical skills and make strategic decisions; and (3) developing practical skills through focused practice and a repeat of the adapted game [49,73,74,76,77].

Numerous researchers have suggested that effective utilization of the TGM in physical education courses can help teachers enhance student motivation for learning [73,74,78]. Hartati et al. [79] conducted a study involving 60 middle school students using the tactical games model (TGM) and established a control group to evaluate the effects. The findings indicated that TGM effectively increased the motivation of these students in a basketball learning context. Similarly, Rosaria Schembri’s study [80] on a handball program for primary students using TGM found an enhanced enjoyment at the session’s conclusion, confirming TGM’s effectiveness in increasing student engagement. However, some studies have presented differing results. Juan M. Garcia-Ceberino and his team analyzed the motivation of primary school students in football and basketball. They considered four dimensions: student gender, movement mode, experience, and teaching method. Their results indicated that the tactical games model did not demonstrate a significant difference in student motivation compared to the direct teaching method [81]. These findings are consistent with the views of many researchers who assert that the effectiveness of game-based teaching approaches varies across educational levels and different sports. This suggests that determining the most effective teaching methods for optimal teaching and learning outcomes necessitates further in-depth research [82,83,84].

Previous research has explored the effects of the TGM in various sports, including basketball [53], volleyball [85], handball [86], and balance [87] activities. These studies have consistently reported the positive impacts of TGM on elementary, middle, and high school students. Further, researchers have observed improvements in aspects such as game performance [88], skill levels [89], and cognitive functions [90] due to TGM. However, when reviewing previous studies, most studies on the tactical games model (TGM) have centered around the K–12 educational level, with relatively few addressing its effects on university students. Tite Juliantine et al. and Dorak Ferudun et al. investigated TGM in university handball programs. Tite Juliantine et al. examined the development of players’ skills, finding a significant enhancement in basic handball skills due to TGM [86]. Dorak Ferudun et al., on the other hand, reported that TGM surpassed the direct teaching model in enhancing students’ game performance [91]. Given the paucity of research on the impact of the tactical games model (TGM) on university students’ motivation in physical education and considering the critical role of motivation in student learning, there is a compelling need for effective teaching methodologies. This study, therefore, aims to assess the influence of TGM on university students’ motivation in basketball courses. It also explores the factors influencing motivation regarding self-determination theory, providing robust data support for teachers and researchers post COVID-19 pandemic. This information will be crucial in devising teaching strategies that enhance student motivation. In addition, to ensure the transparency and quality of our intervention, we utilized “The quality scale of Non-randomized Intervention Studies” (TREND), which is based on the Transparent Reporting of Evaluations with Non-randomized Designs (TREND) statement. This approach was employed to evaluate the intervention, demonstrating our adherence to the TREND statement and confirming the replicability and validity of our methods. For details on the evaluation checklist, please refer to Appendix A.

## 2. Materials and Methods

### 2.1. Study Design

The research design for this study was quasi-experimental, incorporating repeated measures and a non-equivalent control group to ensure comparative analysis. Since the instructional intervention took place in a university setting for a basketball course (where enrolment is predetermined by school administration in a pseudo-random fashion), establishing a non-equivalent control group was crucial. This approach was intended to maintain the study population’s integrity, mirror the natural teaching environment as accurately as possible, and limit influences that could potentially bias the results.

### 2.2. Participants and Procedures

This study involved 164 students (81 females and 83 males) enrolled in sophomore basketball courses at a Henan Province, China, university. An experimental group and a control group were required to adhere to the study’s design. The classes were randomly assigned to these groups to ensure the integrity of the research design [92,93]. The study required full participation in both testing and teaching interventions. However, due to unavoidable confounding influences, 23 students could not complete the testing or fully participate in the study. Consequently, their data were excluded, leaving 141 students in the final analysis. Of these, 68 were in the experimental group (36 males and 32 females), and 73 were in the control group (38 males and 35 females).

The study spanned ten weeks, and experimental and control group participants engaged in a basketball course. To maintain content consistency and teaching quality, the experimental and control groups were taught on predetermined days following the target school’s curriculum plan: the experimental group on Monday and the control group on Wednesday. Evaluations were conducted immediately before and after the instructional intervention. The motivation assessments were administered on the basketball court using mobile phones, with the researcher present to oversee the completion and ensure anonymity. The responses were collected without student queries about the procedure, securing data confidentiality and precision. Furthermore, the intervention lasted eight weeks and adhered closely to the school’s established basketball curriculum to replicate an authentic educational setting. Additionally, the experimental and control groups were taught by two experienced teachers, both with master’s degrees in physical education. One, a professor, has over 30 years of experience in basketball coaching, while the other has over 20 years in physical education and holds a Chinese national basketball referee certificate. Before the study, a researcher pursuing a Ph.D. in sports science, who is well-versed in the tactical games model and has substantial experience applying it, conducted four 60 min training sessions with these teachers. The researcher also actively participated in the study by overseeing the teaching interventions designed according to specific lesson plans for both experimental and control groups. This supervision ensured the interventions aligned closely with the teaching model’s characteristics. Additionally, the researcher monitored the testing process to guarantee the accuracy of the study’s results.

This study, involving non-invasive teaching interventions, complied with the Declaration of Helsinki [94,95] standards for conducting scientific research. Before commencement, the Ethics Committee of Universiti Putra Malaysia approved the study design, methodology, and assessment instruments under the approval code (JKEUPM-2023-488). Additionally, permission was obtained from the sports administration of the target university to conduct the physical education courses.

### 2.3. Intervention Plan

The objective of this study was to explore how the tactical games model (TGM) influences motivation among university students in basketball courses. Considering that most previous research implemented instructional interventions spanning 4 to 12 weeks to study the TGM’s effects on students’ motivation and affective responses, this study adopted an 8-week intervention period. This duration aligns with recommendations from researchers who suggest that mirroring the daily instruction of experimental studies with regular teaching practices can ensure that the intervention mimics the natural educational environment [96]. To maintain the regular teaching schedule of the target school’s basketball courses, the instructional intervention in this study adhered to the school’s existing teaching plan. Each basketball course lasted 90 min, beginning with a mandatory 10 min warm-up session as required by the school. Subsequently, the experimental and control groups proceeded with their respective teaching plans according to the established curriculum. Table 1 provides a detailed breakdown of the TGM course plans and the DIM groups.

Experimental Group

The experimental group was instructed using the tactical games model (TGM) for an 8-week basketball course. This teaching plan was structured around the TGM framework as proposed by J.L. Oslin et al. [73], comprising four key components: (a) an initial modified game (modified game 1); (b) tactical questioning to enhance understanding and application; (c) skill explanations followed by practice sessions; and (d) a concluding modified game (modified game 2) to reinforce learning. During the TGM group instruction, the teacher initially organized the students into three groups. These groups engaged actively in a modified game (modified game 1) designed to introduce them to the game and the day’s learning objectives. Following this, the teacher posed tactical questions about the content, encouraging students to think critically and devise solutions to problems. The teacher then explained the specific skills or tactics and oversaw the students as they practiced, aiming to enhance their understanding and proficiency. The sessions concluded with the groups participating in a second modified game (modified game 2), reinforcing their learning and enabling them to apply the skills in realistic game situations.

Control Group

The control group received basketball instruction using the traditional direct instruction model (DIM). The course followed a structured sequence: (a) review of the previous session’s content; (b) the teacher’s introduction, explanation, and demonstration of new content; (c) student practice in groups; and (d) unstructured free time for activities, stretching, and relaxation. The content taught to the DIM group mirrored that of the TGM group, adhering strictly to the teaching plan without any alterations during the course. Notably, during the free activity period, the teacher arranged no structured games; instead, activities were student-organized.

### 2.4. Instruments

This study utilized the Sport Motivation Scale-II (SMS-II) to assess students’ motivation in basketball courses. In previous studies, the SMS has been widely used to measure self-determined motivation in research participants [97,98,99]. However, as its usage has increased among researchers, some inconsistencies with self-determination theory have been identified in the scale’s items. These discrepancies have raised concerns about potential biases in the results derived from its application. In solving the inconsistencies with self-determination theory, Pelletier, L.G. et al. revised the original Sports Motivation Scale in 2013, creating the Sports Motivation Scale-II (SMS-II). This updated version consists of 18 items distributed across six subscales: non-regulation (AM), external regulation (ER), introjected regulation (IJR), identified regulation (IDR), integrated regulation (IR), and intrinsic regulation (IM). Each item is evaluated using a seven-point Likert scale ranging from 1 (does not correspond at all) to 7 (corresponds completely), where higher scores indicate greater motivation. The SMS-II aligns more closely with self-determination theory and allows for quicker completion, improving the efficiency of data collection [100].

To accommodate the Chinese-speaking participants in this study, the Chinese Sport Motivation Scale-II (CSMS-II) [101], translated and evaluated by Chunxiao Li et al., was employed instead of its English counterpart. The CSMS-II is tailored to measure the motivation of Chinese students in sports contexts, and it exhibited good internal consistency, with Cronbach’s alpha values between 0.69 and 0.76 and composite reliability scores from 0.69 to 0.78. The validity of the CSMS-II was further confirmed through a pilot test, which reported a Cronbach’s alpha of 0.77, indicating reliable internal consistency. (Details are provided in the attached Appendix B.)

### 2.5. Statistical Analysis

The first step in the data analysis was to screen the collected scale assessment scores, removing those that were invalid or did not meet the analytical criteria. Subsequently, descriptive statistics were used to calculate the mean (M), standard deviation (SD), and the difference in means (gain) of the data included in the analysis. For hypothesis testing, the data’s normality was assessed using skewness and kurtosis coefficients, and Levene’s test for homogeneity of variance was employed to evaluate the variance’s homogeneity across datasets. To ascertain the impact of the TGM on university students’ motivation, we analyzed the data using an analysis of variance (ANOVA) within the TGM and DIM groups. Due to the quasi-experimental design with repeated measures in this study, pre-intervention test scores could influence the post-test results after the 8-week intervention period. To ensure the accuracy of the findings, an analysis of covariance (ANCOVA) was employed to assess between-group differences in motivation scores. This analysis also included tests for linear relationships between the variables and checked for homogeneity of regression slopes. For the ANOVA results, eta squared (η²) was used to interpret the effect sizes, which were categorized as small (η² = 0.01), medium (η² = 0.06), and large (η² = 0.14) based on established benchmarks [102]. The ANCOVA results were analyzed using partial eta squared ($\eta_{p}^{2}$), with effect sizes similarly categorized as small ($\eta_{p}^{2}$ = 0.01), medium ($\eta_{p}^{2}$ = 0.06), and large ($\eta_{p}^{2}$ = 0.14) [103]. Data collection and organization were conducted using Excel, while the statistical analysis was performed in SPSS 29.0. The significance level for tests was set at 0.05.

## 3. Results

Pelletier, L.G. et al. suggested using the Self-Determination Index (SDI) to assess participants’ self-determined motivation, where the magnitude and the sign (positive or negative) of the SDI score indicate the strength of motivation [100]. In this study, the SDI was calculated according to the method proposed by J. Mammenga, which involves assigning specific weights to each subscale of the SMS-II: +3 for intrinsic regulation (IM), +2 for integrated regulation (IR), +1 for identified regulation (IDR), −1 for introjected regulation (IJR), −2 for external regulation (ER), and −3 for non-regulation (AM) [104]. These weights were then applied to their respective subscale scores and summed to derive the overall SDI score, indicating the participants’ motivation levels. The SDI score delineates the motivational profile of the participants. A higher SDI scores suggest a greater level of intrinsic motivation, characterized by self-driven interests and enjoyment of the activity. Conversely, lower scores indicate a predominance of extrinsic motivation, where external factors like rewards or pressures motivate the participants or, in some cases, reflect a lack of motivation altogether.

Table 2 outlines the descriptive statistics (mean values, standard deviations, and mean growth) of the CSMS-II scores for both groups, captured before and after the experimental intervention.

The results indicated that students in the TGM group experienced a significant improvement in motivation (SDI gain = 11.9). Conversely, students in the DIM group exhibited a decline in motivation (SDI gain = −10.41). Further analysis revealed that TGM positively influenced the subscales related to intrinsic motivation (IM gain = 2.34; IR gain = 2.04; IDR gain = 1.39). In contrast, the DIM group did not positively impact intrinsic motivation, showing declines across the subscales (IM gain = −0.88; IR gain = −0.04; IDR = −0.04).

Motivation scores for students in both the TGM and DIM groups were analyzed using ANOVA to determine the effects of the TGM on university students’ motivation. Additionally, ANCOVA analyses were utilized to compare the differential effects of TGM and DIM on student motivation, providing a more comprehensive understanding of TGM’s efficacy.

Table 3 displays the outcomes of normality testing and homogeneity of variance testing for the pre- and post-teaching intervention motivation scores of students enrolled in the TGM and DIM groups.

The results indicated skewness values (−0.268 to 1.163) and kurtosis values (−1.203 to 1.055) for the SDI and subscale scores in both TGM and DIM groups. As these values fall within the acceptable range of −2 to 2, the data can be assumed to conform to a normal distribution. Additionally, the variance chi-square test for SDI showed no significant differences between groups, with TGM (F = 0.351, *p* = 0.554 > 0.05) and DIM (F = 0.183, *p* = 0.669 > 0.05). Similarly, the subscale scores also showed no significant pre- and post-intervention differences, indicating that the motivation scores meet the assumptions required for conducting ANOVA analysis.

Table 4 displays the ANOVA results for the motivation scores of students in both the TGM and DIM groups (mean, standard deviation, statistical significance, and effect size).

The results indicate significant changes in motivation scores postintervention for both the TGM and DIM groups. Specifically, the TGM group showed significant improvements in SDI scores (F = 6.949, *p* = 0.009 < 0.05), as did the DIM group (F = 4.681, *p* = 0.032 < 0.05). For the CSMS-II subscales, the TGM group exhibited significant gains in intrinsic motivation measures such as IM (F = 13.698, *p* < 0.001), IR (F = 10.546, *p* = 0.001 < 0.05), and IDR (F = 4.283, *p* = 0.040 < 0.05). However, significant changes were observed in the extrinsic motivation subscales: ER (F = 7.205, *p* = 0.008 < 0.05) and AM (F = 4.125, *p* = 0.044 < 0.05). Conversely, the DIM group did not demonstrate significant differences in intrinsic motivation subscales: IM (F = 1.794, *p* = 0.183 > 0.05), IR (F = 0.003, *p* = 0.955 > 0.05), IDR (F = 0.077, *p* = 0.782 > 0.05), and IJR (F = 0.366, *p* = 0.546 > 0.05).

To more precisely assess the effects of the TGM on university students’ motivation, we established a control group that followed a traditional teaching method. Given the potential influence of initial motivation assessment scores on subsequent results, we employed ANCOVA. This method helped remove the influence of pre-existing differences, ensuring a more precise analysis of the intervention’s actual effects. Initially, we analyzed the motivation scores of both groups following the instructional intervention. The analysis confirmed a linear regression relationship between the pre-and post-intervention scores for both the TGM and DIM groups, validating the assumptions necessary for the ANCOVA analysis (as shown in Table 5). Homogeneity tests for regression slopes in SDI (F = 0.865, *p* = 0.354 > 0.05), IR (F = 1.010, *p* = 0.317 > 0.05), IDR (F = 0.038, *p* = 0.845 > 0.05), ER (F = 0.037, *p* = 0.847 > 0.05), and AM (F = 1.491, *p* = 0.224 > 0.05) indicated no significant differences, thereby meeting the criteria for conducting ANCOVA. However, disparities in the regression slopes for the IM (F = 7.736, *p* = 0.006 < 0.05) and IJR (F = 6.351, *p* = 0.013 < 0.05) subscales suggested unequal effects. Consequently, non-parametric ANCOVA analyses were employed to enhance the precision of the results. Table 5 shows the linear regression relationship and the homogeneity test of the regression for the motivation score data between the TGM and DIM groups after the teaching intervention.

Table 6 presents the ANCOVA analysis results for the motivation scores of the TGM and DIM groups following the instructional intervention.

The results indicated a more favorable effect of TGM on students’ motivation than DIM. Significant differences were observed in the overall SDI scores (F = 65.186, *p* < 0.001) and across various subscales: IM (F = 5.570, *p* < 0.001), IR (F = 11.171, *p* = 0.001 < 0.05), IDR (F = 8.124, *p* = 0.005 < 0.05), ER (F = 4.689, *p* = 0.032 < 0.05), and AM (F = 12.565, *p* < 0.001). The only exception was the IJR subscale, with no significant difference (F = 0.550, *p* = 0.583 > 0.05).

## 4. Discussion

The objective of this study was to examine the effects of the tactical games model (TGM) on enhancing university students’ motivation in a basketball course. We conducted a teaching experiment as the intervention, with student motivation assessed using the Sports Motivation Scale-II (SMS-II). Results indicated that TGM effectively enhances students’ motivation. Specifically, instructors who employ TGM in university basketball courses can significantly boost students’ intrinsic motivation, achieving superior outcomes compared to traditional teaching methods. Previous studies have consistently highlighted students’ motivation as a critical concern in teaching physical education. Grounded in self-determination theory, it is posited that intrinsic motivation is a crucial driver of students’ engagement in sports and physical activities. We believe that students participating in basketball courses voluntarily leads to superior performance and learning outcomes compared to participation driven by external motivators, such as grades or other incentives. Therefore, the analysis in this study focused on evaluating students’ motivation within a basketball course, utilizing the Self-Determination Index (SDI) and the six intrinsic and extrinsic motivation factors proposed by the Sports Motivation Scale-II (SMS-II).

The analysis results of the Self-Determination Index (SDI) indicated significant differences in motivation among students in both TGM and DIM groups, with ANOVA showing notable changes pre- and postintervention: TGM (F = 6.949; *p* = 0.009; η² = 0.049) and DIM (F = 4.681; *p* = 0.032; η² = 0.031). Descriptive statistics further underscored these findings, showing a notable mean increase in SDI for the TGM group in contrast to a decrease for the DIM group. Moreover, TGM demonstrated a superior effect on enhancing students’ motivation compared to DIM (F = 65.186; *p* < 0.001; $\eta_{p}^{2}$ = 0.66). This study’s findings corroborate those of Hartati et al. [79], who reported a significant difference in motivation between students taught using the TGM and those in the control group. TGM notably enhanced students’ motivation to learn basketball. Similarly, our observations during the instructional intervention revealed that students in the TGM group exhibited a markedly higher motivation to participate in the basketball course. In contrast, students in the DIM group displayed negative attitudes toward participation, with some expressing a desire to switch courses after the experience. This aligns with the observed decline in motivation scores for the DIM group, which decreased significantly postintervention (gain = −10.41). We analyzed these results through the lens of both intrinsic and extrinsic motivation to understand the underlying factors.

In assessing intrinsic motivation, the Sports Motivation Scale-II (SMS-II) assessed intrinsic motivation through three subscales: intrinsic regulation (IM), integrated regulation (IR), and identified regulation (IDR). Students in the TGM group exhibited significant gains in these areas, with IM (gain = 2.34; η² = 0.093; medium effect), IR (gain = 2.04; η² = 0.073; medium effect), and IDR (gain = 1.39; η² = 0.031; small effect). We attribute the success of the TGM in enhancing intrinsic motivation to its structured teaching plan, which incorporates modified games at both the beginning and end of the basketball course. This format makes learning enjoyable and allows students to experience the thrill of teamwork and victory in basketball. Furthermore, it provides ample opportunities for students to immediately apply the skills and tactics they have learned, thus reinforcing their mastery and satisfaction from achieving tangible learning outcomes. By integrating engaging game units into the curriculum, TGM maximizes the intrinsic allure of sports like basketball, motivating students to engage more actively in the course. Our results support previous studies that found TGM significantly improves students’ enjoyment and pleasure during physical education courses. TGM’s focus on engaging game units makes learning more enjoyable and increases students’ enthusiasm for participating in physical education activities [80,105,106,107]. Contrary to the TGM group, intrinsic motivation scores for students in the DIM group decreased across three subscales: IM (gain = −0.88, η² = 0.012), IR (gain = −0.04, η² < 0.001), and IDR (gain = −0.18, η² = 0.001). This decrease may be linked to the traditional teaching methods, characterized by a teacher-centric approach with little student autonomy. This method typically focuses on skill acquisition through repetitive practice, which may become monotonous and uninspiring for students. Moreover, while the course design included free activity sessions intended for the practical application of skills in real-game settings, most students opted out of active participation, choosing instead to rest, further diminishing opportunities for experiencing the competitive thrill and collaborative joy of basketball. Furthermore, the ANCOVA conducted on the motivation scores between the two groups revealed that the TGM enhanced students’ intrinsic motivation more than the DIM. Significant improvements were noted in IM (F = −5.570; *p* = 0.000; $\eta_{p}^{2}$ = 0.50; high effect), IR (F = 11.171; *p* = 0.001; $\eta_{p}^{2}$ = 0.47; high effect), and IDR (F = 8.124; *p* = 0.005; $\eta_{p}^{2}$ = 0.43; high effect).

The analysis of extrinsic motivation also yielded noteworthy results. In the subscales of introjected regulation (IJR) and external regulation (ER), increases were observed in both the TGM and DIM groups. Specifically, the TGM group saw gains in IJR (gain = 0.66; η² = 0.008; small effect) and ER (gain = 0.45; η² = 0.005; small effect). In contrast, the DIM group experienced more pronounced increases with IJR (gain = 0.40; η² = 0.003; small effect) and ER (gain = 1.55; η² = 0.048; small effect), indicating a more substantial influence of extrinsic motivators on the DIM group. This indicates that the observed decrease in motivation among students in both the TGM and DIM groups after the teaching intervention may be attributed to internal and external pressures. Students were more motivated by the prospect of achieving a better final grade than by the desire to enhance their skills or achieve learning outcomes. There was a noticeable lack of enthusiasm for active participation in the course. Additionally, some students reported choosing the basketball course because they were more familiar with the sport than other options, believing it would help them pass the final test more efficiently. Others saw the course as an opportunity to engage in physical activity to lose weight.

Furthermore, in the non-regulation (AM) subscale, the TGM group outperformed the DIM group, as evidenced by significant differences in the post-intervention ANCOVA analysis (F = 12.565; *p* < 0.001; $\eta_{p}^{2}$ = 0.53). Despite decreased scores, as shown in the descriptive statistics (gain = −0.32, η² = 0.002), the TGM approach increased students’ interest in learning basketball. The competitive and problem-solving elements introduced by TGM, including strategically designed matches and tactical questioning, likely enhanced students’ curiosity and engagement, motivating them to participate and actively address challenges within the games. At the same time, during the second modified game, we observed a notable increase in enthusiasm among the participants. The students displayed a heightened sense of pride and satisfaction in their expressions when they successfully applied their newly acquired skills and tactics to score points or defensively block their opponents. These observations suggest that the TGM likely enhances extrinsic motivation by influencing non-regulation aspects of motivation. In contrast, students in the DIM group showed more significant improvements in extrinsic motivation scores, and there was a noticeable decline in their interest in basketball following the intervention. This discrepancy suggests a potential issue with traditional teaching methods, underscoring the importance for physical education teachers to consider how extrinsic motivation factors influence student engagement and motivation in sports programs.

In addition, this study contributes to filling a research gap in applying the tactical games model (TGM) in university environments. Post COVID-19, it offers a fresh perspective and innovative methods for university physical education, providing educators with practical strategies to increase student motivation in basketball courses. The findings indicate that the use of modified games within the TGM program significantly enhanced students’ intrinsic motivation and enjoyment, highlighting the importance of intrinsic motivation in promoting student engagement. Research suggests that improving the teaching and learning environment can lead to superior educational outcomes [108,109], underscoring the significance of promoting TGM in university physical education programs.

## 5. Research Strengths and Limitations

The purpose of this study was to assess the impact of the tactical games model (TGM) on university students’ motivation to engage in basketball courses. The findings strongly support using TGM as an effective means to boost student motivation and advocate for the broader adoption of game-centered teaching methods in university settings. Additionally, this research underscores the importance of exploring new teaching methodologies to improve student motivation, particularly in recovering from COVID-19 pandemic disruptions. Consistent with prior research, our findings emphasize the critical role of addressing intrinsic and extrinsic motivations in learning, helping educators tackle contemporary educational challenges and improve student learning environments.

Meanwhile, this study has several limitations worth noting. First, our sampling method involved selecting four classes from the existing basketball program, which does not adhere to actual random sampling techniques. While this approach allowed us to maintain a natural teaching environment and did not disrupt the target schools’ regular schedule and progression, it limits the generalizability of our findings. As such, the results may not fully represent broader populations or different educational contexts.

A further limitation of this study is the specificity of the sport involved. Basketball was chosen due to its popularity among Chinese universities and its favorability among students. However, different sports may have varied psychological impacts on students. Although Stephen A. Mitchell et al. [73]. suggested that the benefits and learning outcomes of using TGM are transferable across different game units, further research is needed to explore the impact of TGM on university students engaged in various sports to validate and possibly expand upon these findings.

Additionally, this study did not account for the student’s personal perceptions or subjective experiences with the tactical games model (TGM), focusing instead on an objective analysis from a third-party perspective; this represents a significant limitation. Incorporating qualitative research methods such as interviews to capture students’ subjective views and feelings could provide a more comprehensive understanding of the impact of TGM. Future research should aim to explore these subjective experiences to enhance the foundational evidence of the study’s findings.

## 6. Conclusions

This study contributes to the knowledge of pedagogical methods in university physical education by highlighting the tactical games model (TGM) as an effective strategy for increasing student motivation post COVID-19 pandemic. TGM has proven to engage students more actively in basketball courses and promote broader participation in physical activities, aligning with educational objectives in physical health. Furthermore, this research fills a critical gap, extending the application of game-centered teaching strategies, which are traditionally researched within the K–12 context, to university settings. The physical and mental health impacts of such pedagogical approaches on university students also merit further exploration and consideration by educators and researchers. The results from this study indicated that students had significant variations in their motivation after participating in an eight-week teaching intervention. Specifically, TGM significantly enhanced students’ self-determined motivation, intrinsic motivation, and extrinsic motivation compared to traditional teaching methods. These findings underscore TGM’s potential as a superior pedagogical strategy for increasing motivation in university physical education courses.

This study’s results not only corroborate previous research that effective teaching methods can enhance students’ motivation and enrich their learning experiences in physical education, but they also provide a foundation for further academic inquiry [74,110]. Additionally, these results serve as a catalyst for university physical education researchers to pursue more comprehensive studies on student motivation, particularly through the application of game-centered approaches like TGM, in universities. This research not only contributes to the theoretical development of teaching methods but also aims to elevate the standard of physical education teaching and learning at the university level.

## Figures and Tables

**Table 1:** TGM Group and DIM Group Course Plans.

Session	Content	Tactical Games Model Group		DIM	
		Schedule	Time	Schedule	Time
1st	Jump Shot	Warm-up	10 min	Warm-up	10 min
		Introduction and Engagement in 3v3 Shooting Game 1	20 min	Review the Content Learned in the Previous Lesson	15 min
		Inquiry on Tactical Questions, Demonstration, and Explanation of the Jump Shot Technique (Q: What actions did you take to score under tight defense?)	20 min	Introduce, Demonstrate, and Break Down the Jump Shot Technique	15 min
		Pairing Students into Groups of Two for 1v1 Practice	20 min	Students Practice the Jump Shot Technique Individually	20 min
		Conducting 3v3 Game 2	20 min	Free Activity/Stretching and Relaxation	30 min
2nd	Cut Technique	Warm-up	10 min	Warm-up	10 min
		Introduction and Engagement in 3v3 Passing Drills Game 1	20 min	Review the Jump Shot Learned in the Previous Lesson	15 min
		Inquiry on Tactical Questions, Demonstration, and Explanation of the Cutting Movement (Q: What is the objective of this game? How did you achieve that? What do you need to consider before making a cut?)	20 min	Introduce, Demonstrate, and Break Down the Cutting Technique	15 min
		Grouping Students into Teams of Three for 2v1 Cutting Practice	20 min	Divide Students into Two Groups, Each Group Practices the Cutting Technique Separately	20 min
		Conducting 3v3 Game 2	20 min	Free Activity/Stretching and Relaxation	30 min
3rd	Pass and Cut Coordination	Warm-up	10 min	Warm-up	10 min
		Introduction and Engagement in 3v3 Game 1	20 min	Review the Cutting Technique Learned in the Previous Lesson	15 min
		Inquiry on Tactical Questions, Demonstration, and Explanation of Pass and Cut Coordination (Q: As an off-ball player, how did you get open within the key? What did you do to prevent the defender from getting between you and the ball? What do you need to consider before going for a layup to score?)	20 min	Introduce, Demonstrate, and Break Down the Pass and Cut Coordination	15 min
		Grouping Students into Teams of Four for 2v2 Pass and Cut Practice	20 min	Pair Students into Groups of Two for Practice on Pass and Cut Coordination	20 min
		Conducting 3v3 Game 2	20 min	Free Activity/Stretching and Relaxation	30 min
4th	Screen Play Coordination	Warm-up	10 min	Warm-up	10 min
		Introduction and Engagement in 3v3 Game 1	20 min	Review the Pass and Cut Coordination Learned in the Previous Lesson	15 min
		Inquiry on Tactical Questions, Demonstration, and Explanation of Screen Play Coordination (Q: How did you manage to get the ball-20min handler an open shot? What is the best body position for a player to set an effective screen? What is the best way for the ball-handler to use a screen?)	20 min	Introduce, Demonstrate, and Break Down the Screen Play Coordination	15 min
		Grouping Students into Teams of Three for 2v1 Screen Practice	20 min	Group Students into Teams of Three to Practice Screen Play Coordination	20 min
		Conducting 3v3 Game 2	20 min	Free Activity/Stretching and Relaxation	30 min
5th	Pick and Roll Coordination	Warm-up	10 min	Warm-up	10 min
		Introduction and Engagement in 3v3 Game 1	20 min	Review the Drive and Dish Coordination Learned in the Previous Lesson	15 min
		Inquiry on Tactical Questions, Demonstration, and Explanation of Pick and Roll Coordination (Q: As an off-ball player, how did you get open within the key? How did you determine where to set the screen? What should you do when your teammate approaches your defender trying to set a screen for you?)	20 min	Introduce, Demonstrate, and Break Down the Pick and Roll Coordination	15 min
		Grouping Students into Teams of Five for 3v2 Pick and Roll Practice	20 min	Group Students into Teams of Four to Practice Pick and Roll Coordination	20 min
		Conducting Game 2	20 min	Free Activity/Stretching and Relaxation	30 min
6th	Rebounding	Warm-up	10 min	Warm-up	10 min
		Introduction and Engagement in 3v3 Rebounding Game 1	20 min	Review the Pick and Roll Coordination Learned in the Previous Lesson	15 min
		Inquiry on Tactical Questions, Demonstration, and Explanation of Rebounding (Q: As an off-ball offensive player, what actions did you take after your teammate’s shot to get a rebound? As an off-ball defensive player, what actions did you take after the opponent’s shot to get a rebound? As the shooter, what actions did you take after your shot to get a rebound?)	20 min	Introduce, Demonstrate, and Break Down the Rebounding Technique	15 min
		Grouping Students into Teams of Four for 2v2 Rebounding Drills	20 min	Pair Students into Groups of Two to Practice the Rebounding Technique	20 min
		Conducting 3v3 Game 2	20 min	Free Activity/Stretching and Relaxation	30 min
7th	Defending Off-Ball Players	Warm-up	10 min	Warm-up	10 min
		Introduction and Engagement in 3v3 Game 1	20 min	Review the Rebounding Learned in the Previous Lesson	15 min
		Inquiry on Tactical Questions, Demonstration, and Explanation of Defending Off-Ball Players (Q: What did you do to prevent the opponent from scoring? Which defensive positions or movements were most effective in disrupting the opponent’s offense and preventing them from scoring? As the shooter, what actions did you take after your shot to get a rebound?)	20 min	Introduce, Demonstrate, and Break Down the Technique for Defending Off-Ball Players	15 min
		Pairing Students into Groups of Two for Practice on Defending Off-Ball Players	20 min	Group Students into Teams of Six to Practice Defending Off-Ball Players	20 min
		Conducting 3v3 Game 2	20 min	Free Activity/Stretching and Relaxation	30 min
8th	Defending On-Ball Players	Warm-up	10 min	Warm-up	10 min
		Introduction and Engagement in 3v3 Game 1	20 min	Review Defending Off-Ball Players Learned in the Previous Lesson	15 min
		Inquiry on Tactical Questions, Demonstration, and Explanation of Defending On-Ball Players (Q: What did you do to prevent the opponent from scoring? Which defensive positions or movements were most effective in disrupting the opponent’s offense and preventing them from scoring? As the shooter, what actions did you take after your shot to get a rebound?)	20 min	Introduce, Demonstrate, and Break Down the Technique for Defending On-Ball Players	15 min
		Grouping Students into Teams of Four for 2v2 Practice on Defending On-Ball Players	20 min	Group Students into Teams of Six to Practice Defending On-Ball Players	20 min
		Conducting 3v3 Game 2	20 min	Free Activity/Stretching and Relaxation	30 min

**Table 2:** CSMS-II Descriptive Statistics.

Variable	TGM Group (n = 68)					DIM Group (n = 73)				
	Pretest		Posttest		Gain	Pretest		Posttest		Gain
	*Mean*	*SD*	*Mean*	*SD*		*Mean*	*SD*	*Mean*	*SD*	
IM	13.76	4.03	16.10	3.30	2.34	15.48	3.88	14.60	4.03	−0.88
IR	11.56	3.83	13.60	3.50	2.04	12.85	4.58	12.81	4.20	−0.04
IDR	14.37	4.12	15.76	3.74	1.39	15.37	3.87	15.19	3.89	−0.18
IJR	8.78	3.78	9.44	3.49	0.66	9.49	3.61	9.89	4.29	0.40
ER	5.81	3.19	6.26	3.47	0.45	6.00	3.13	7.55	3.80	1.55
AM	7.54	3.49	7.22	3.37	−0.32	7.33	3.79	8.67	4.18	1.34
SDI	35.75	27.03	47.65	25.58	11.9	44.03	29.35	33.62	28.78	−10.41

Note: TGM, tactical games model; DIM, direct instruction model; SD, standard deviation; IM, intrinsic regulation; IR, integrated regulation; IDR, identified regulation; IJR, introjected regulation; ER, external regulation; AM, non-regulation; SDI, Self-Determination Index.

**Table 3:** Normality tests and homogeneity of variance tests for motivation scores in the TGM and DIM groups.

Variable	TGM Group						DIM Group					
	Pretest		Posttest		F *	Sig.	Pretest		Posttest		F *	Sig.
	Sk	Ku	Sk	Ku			Sk	Ku	Sk	Ku		
IM	−0.161	−0.366	0.119	−1.203	1.183	0.279	−0.241	−0.901	−0.231	−0.248	0.011	0.917
IR	−0.121	−0.166	0.276	−0.048	0.535	0.466	0.180	−0.616	0.126	−0.329	0.597	0.441
IDR	−0.137	−0.634	−0.166	−0.787	0.224	0.637	−0.138	−0.626	−0.268	−0.095	0.074	0.786
IJR	0.639	−0.216	0.129	−0.787	0.474	0.492	0.319	0.046	0.584	0.079	1.135	0.288
ER	0.992	−0.092	1.163	0.895	0.130	0.719	1.111	1.055	0.837	0.336	3.369	0.068
AM	0.219	−1.034	0.311	−1.076	0.007	0.934	0.576	−0.456	0.457	−0.253	0.442	0.507
SDI	0.105	−0.839	0.098	−0.666	0.351	0.554	−0.202	−0.709	0.089	−0.531	0.183	0.669

* Levene’s homogeneity. Note: TGM, tactical games model; DIM, direct instruction model; Sk, skewness; Ku, kurtosis; IM, intrinsic regulation; IR, integrated regulation; IDR, identified regulation; IJR, introjected regulation; ER, external regulation; AM, non-regulation; SDI, Self-Determination Index.

**Table 4:** ANOVA Analysis.

Variable	TGM Group					DIM Group				
	M (SD) Pre	M (SD) Post	*F*	Sig.	η²	M (SD) Pre	M (SD) Post	*F*	Sig.	η²
IM	13.76(4.03)	16.10(3.30)	13.698	<0.001	0.093	15.48(3.88)	14.60(4.03)	1.794	0.183	0.012
IR	11.56(3.83)	13.60(3.50)	10.546	0.001	0.073	12.85(4.58)	12.81(4.20)	0.003	0.955	<0.001
IDR	14.37(4.12)	15.76(3.74)	4.283	0.040	0.031	15.37(3.87)	15.19(3.89)	0.077	0.782	0.001
IJR	8.78(3.78)	9.44(3.49)	1.126	0.291	0.008	9.49(3.61)	9.89(4.29)	0.366	0.546	0.003
ER	5.81(3.19)	6.26(3.47)	0.636	0.426	0.005	6.00(3.13)	7.55(3.80)	7.205	0.008	0.048
AM	7.54(3.49)	7.22(3.37)	0.302	0.583	0.002	7.33(3.79)	8.67(4.18)	4.125	0.044	0.028
SDI	35.75(27.03)	47.65(25.58)	6.949	0.009	0.049	44.03(29.35)	33.62(28.78)	4.681	0.032	0.031

Note: TGM, tactical games model; DIM, direct instruction model; M, mean, SD, standard deviation; IM, intrinsic regulation; IR, integrated regulation; IDR, identified regulation; IJR, introjected regulation; ER, external regulation; AM, non-regulation; SDI, Self-Determination Index.

**Table 5:** Homogeneity of the linear relationships and regression slopes for motivation scores between TGM and DIM groups.

Variable	Linear Relationships Tests			Homogeneity of Regression Slopes		
	Type III Sum of Squares	*F*	Sig.	Type III Sum of Squares	*F*	Sig.
IM	908.765	144.205	<0.001	49.653	7.736	0.006
IR	989.522	124.358	<0.001	8.033	1.010	0.317
IDR	1075.943	156.031	<0.001	0.267	0.038	0.845
IJR	729.371	71.460	<0.001	62.406	6.351	0.013
ER	409.425	39.239	<0.001	0.391	0.037	0.847
AM	1019.915	140.483	<0.001	10.789	1.491	0.224
SDI	72,221.082	318.626	<0.001	196.236	0.865	0.354

Note: IM, intrinsic regulation; IR, integrated regulation; IDR, identified regulation; IJR, introjected regulation; ER, external regulation; AM, non-regulation; SDI, Self-Determination Index.

**Table 6:** ANCOVA Analysis.

Variable	TGM		DIM		*F*	Sig.	$\eta_{p}^{2}$
	*MD*	*SD*	*MD*	*SD*			
IM	2.34	3.212	−0.88	2.571	−5.570 ^a^	0.000 ^a^	0.50
IR	2.04	3.239	−0.04	3.199	11.171	0.001	0.47
IDR	1.40	2.771	−0.18	2.983	8.124	0.005	0.43
IJR	0.66	3.724	0.40	3.235	−0.550 ^a^	0.583 ^a^	0.35
ER	0.46	3.289	1.55	3.734	4.689	0.032	0.24
AM	−0.32	2.751	1.34	2.931	12.565	<0.001	0.53
SDI	11.90	16.598	−10.41	15.352	65.186	<0.001	0.66

^a^ Non-parametric ANCOVA. Note: TGM, tactical games model; DIM, direct instruction model; MD, mean difference; SD, standard deviation; IM, intrinsic regulation; IR, integrated regulation; IDR, identified regulation; IJR, introjected regulation; ER, external regulation; AM, non-regulation; SDI, Self-Determination Index.

## Data Availability

Data are contained within the article.
